# Education pathways to the labour market for 16-year-olds who struggle to achieve maths and English in General Certificate of Secondary Education

**DOI:** 10.23889/ijpds.v8i2.2306

**Published:** 2023-09-14

**Authors:** Emma Gorman, Dave Thomson, Peter Urwin, Zhang Min

**Affiliations:** 1 University of Westminster, London, United Kingdom; 2 FFT Datalab, London, United Kingdom

We examine the post age-16 educational pathways taken by the 44% of young people who do not gain “good” grades in English and Maths at age 16 years. We then assess the causal effects of attending General Further Education (GFE) colleges on education and labour market outcomes for this group.

We use the Longitudinal Education Outcomes dataset, which comprises linked administrative education, employment and income records for the population of English school pupils aged 16 in 2011. To summarise complex post-16 education trajectories, we present Sankey charts stratified by indicators of disadvantage. We study the effects of attending GFE at age 17 on whether a pupil gains a Level 3 qualification by age 19, and their earnings and employment status at age 24. To estimate a causal impact, we use distance from home to the closest GFE college as an instrumental variable, controlling for a rich set of background characteristics.

Our graphical results highlight the complexity of post-16 educational pathways and transitions, which are differentiated by disadvantage. Over 50% have GFE as their first post-16 destination. Results from instrumental variable analyses show a positive association between attending GFE and gaining a Level 3 qualification by age 19, among pupils who do not gain a “good” pass in the General Certificate of Secondary Education (GCSE) in either English and/or Maths. Restricting analyses to the bottom of the distribution – those who gain an E, F or G grade in both English and Maths - we do not detect an impact of GFE on qualifications at age 19. Among both subgroups, we do not detect any impact of attending GFE on earnings and employment at age 24 years.

While the post-16 pathways taken by disadvantaged, lower-attaining pupils do increase qualification attainment for some, the value these have in the labour market appears limited. These results may indicate the importance of “soft-skills” and early employment experiences for this subgroup of lower-attainers.

